# Collapsing glomerulopathy in an HIV-positive patient in a low-incidence belt

**DOI:** 10.4103/0971-4065.73451

**Published:** 2010-10

**Authors:** I. Naaz, R. Wani, M. S. Najar, K. Banday, K. M. Baba, H. Jeelani

**Affiliations:** Department of Pathology, Sher-I-Kashmir Institute of Medical Sciences, Srinagar, Jammu & Kashmir, India; 1 Department of Nephrology, Sher-I-Kashmir Institute of Medical Sciences, Srinagar, Jammu & Kashmir, India

**Keywords:** Collapsing glomerulopathy, focal segmental glomerulosclerosis, HIV, HIV-associated nephropathy

## Abstract

Human immunodeficiency virus (HIV) involves glomerular, tubulointerstitial, and vascular compartments of the kidney. The most common glomerular lesion is HIV-associated focal segmental glomerulosclerosis (FSGS) and related mesangiopathies collectively termed HIV-associated nephropathy (HIVAN). A variety of immune-complex mediated glomerular diseases such as membranoproliferative glomerulonephritis (MPGN), IgA nephropathy, and lupus-like glomerulonephritis also occur. HIVAN is restricted to patients presenting with proteinuria and progressive reduction of renal function and with distinctive but not pathognomonic pathology (FSGS often coexisting with glomerular collapse and tubular microcystic dilatations). The worldwide incidence of collapsing glomerulopathy (CG) in HIV-positive patients is high in Americans. But in India and other Asian countries, other forms of kidney diseases are more commonly seen. We report the first case of CG in the state of Jammu and Kashmir which also happens to be a very low incidence belt for HIV.

## Introduction

Collapsing glomerulopathy (CG) is a morphologic variant of focal segmental glomerulosclerosis (FSGS) characterized by segmental and most often global collapse of the glomerular capillaries, marked hypertrophy and hyperplasia of podocytes, and severe tubulointerstitial disease with microcystic dilatation of renal tubules.[1] It is the most common form of HIV-associated nephropathy (HIVAN), though other forms of kidney disease may also occur with HIV.[2] Regardless of the underlying histology, renal disease in HIV-positive patients is associated with an increased risk of death.[3] The available stu-dies have shown that HIVAN is more common in the USA and UK and mesan-gioproliferative glomerulonephritis, membra-nous nephropathy, and membranoprolife-rative glomerulonephritis are more common in Italy, Thailand, and in northern India.[4] While the exact cause for this discrepancy is not entirely clear, racial pre-disposition, viral genotype, and other immu-nomodulatory host susceptibility factors may play a role or could be simply because of dearth of data in the said population. Here we report a case of CG in an HIV-positive patient in a low-incidence belt, Kashmir. This is the first such case from this part of the country.

## Case Report

A 60-year-old homosexual man was admitted with complaints of generalized edema and pallor. There was no history of any drug addiction – oral or intravenous. General physical examination revealed temperature of 99°F, pulse rate of 80/min, respiratory rate of 14/min, and BP of 130/80 mmHg. Patient had pallor and generalized edema. Chest examination revealed decreased breath sounds in the right infrascapular area. Abdominal wall edema was seen and patient had ascites too. CVS and CNS examination was normal.

Laboratory tests revealed a white cell count of 11,000/mm^3^ (69% neutrophils, 20% lymphocytes, 11% monocytes), a hemoglobin concentration of 8.7 g/dL, a platelet count of 164,000/mm^3^, serum urea and creatinine concentration of 67 and 2.8 mg/dL, respectively, serum albumin concentration of 1.8 g/dL, and total protein of 4.3g/dL. Liver enzymes were normal. Pulmonary function tests were within normal limits. Urine examination showed the presence of albumin and casts, RBCs 12–15, polymorphs 8–10 per high power field, and no sugar. And 24-h urinary protein was 5 g. ultrasound of abdomen revealed enlarged kidneys with loss of corticomedullary differentiation. Chest X-ray revealed right-sided pleural effusion. ECG was within normal limits. The patient was then evaluated for HIV, hepatitis B surface antigen (HBsAg), and hepatitis C virus (HCV) infection. An enzyme-linked immunosorbent assay (ELISA) and a Western blot assay for HIV-1 antibodies was positive. CD4 counts were 192/*μ*L and HIV viral load was found to be >4000/mL. Tests for HBV, HCV, CMV, and HTLV-1 were negative. Serum urea and creatinine rose to 74 and 3.3 g/dL after admission.

**Figure 1:**
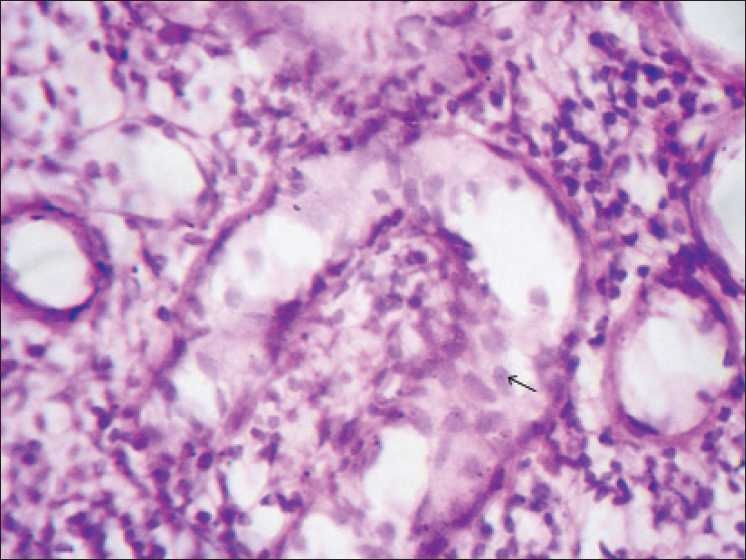
Photomicrograph showing collapsed glomerular tuft and proliferation of visceral epithelial cells (arrows) (H and E stain, ×40)

**Figure 2:**
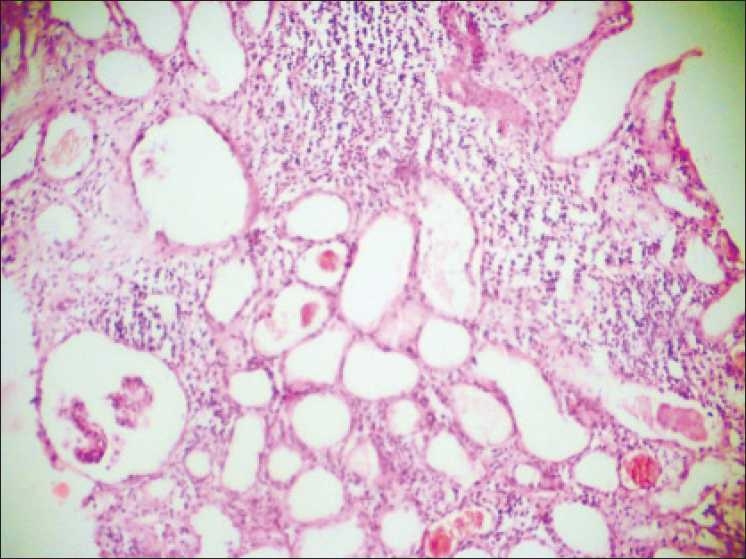
Photomicrograph showing collapsed glomerulus, severe interstitial injury with hyaline and haemorrhagic casts within the dilated tubules. (H and E stain, ×40)

**Figure 3:**
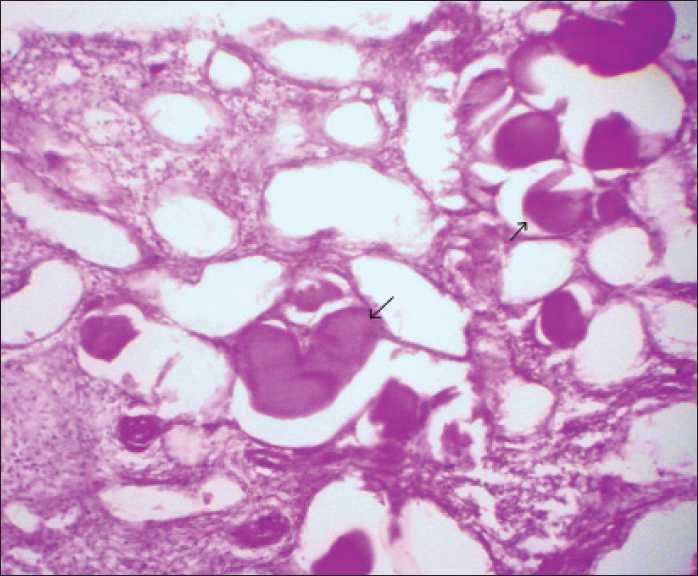
Photomicrograph showing PAS-positive material within the dilated tubules (arrows) (PAS stain, ×40)

Renal biopsy was done. Light microscopic examination of the renal biopsy revealed nine glomeruli with five glomeruli showing global collapse of the capillary tuft, obliteration of the capillary lumens, increase in the mesangial matrix, expansion of the urinary space, and focal glomerulosclerosis. There was proliferation of visceral epithelial cells and showed cytoplasmic vacuolization, associated with microcystic dilatation of tubules, focal tubular atrophy, and the presence of large dense periodic acid-Schiff (PAS)-positive tubular casts. Interstitium showed severe inflammatory infiltration, comprising of both acute and chronic inflammatory cells, and interstitial fibrosis resulting in destruction of tubules at places. Vessels showed features of vasculitis [Figures 1 and 2]. PAS staining showed areas of focal glomerulosclerosis and casts within the tubules [Figure 3]. Immunofluorescence showed only weak nonspecific glomerular IgM deposits. The diagnosis of FSGS collapsing variant was arrived at.

In the hospital, patient was managed for generalized anasarca and anemia while being investigated. He was put on highly active antiretroviral therapy (HAART), ACE inhibitors, and corticosteroids. At 1 month follow-up, he had some improvement in kidney function tests with creatinine dropping to 2.1 mg/dL and 24-h urine protein dropping to 3.6 g/day. Volume overload state had regressed and patient had only edema feet. He failed to collect his course of HAART at third month from ART center located in our institution.

## Discussion

With the advent of HAART, the incidence of opportunistic infections has declined substantially, and cardiovascular, liver, and renal diseases have emerged as major causes of morbidity and mortality in individuals with HIV. Acute renal failure is common in HIV-infected patients and is associated with acute infection and medication-related nephrotoxicity. HIVAN is the most common cause of chronic kidney disease in HIV-positive African American populations and may respond to HAART. Other important HIV-associated renal diseases include HIV-immune complex kidney diseases and thrombotic microangiopathy.[5]

HIVAN develops as a result of HIV gene expression in renal tissue that results in cytopathic effects via the viral transcrip-tional factors or cytokines. Clinical presen-tation typically includes proteinuria without significant hematuria on urinalysis, rapidly progressive renal insufficiency, and large echogenic kidneys.[6] Albuminuria correlates in-versely with the CD4-cell counts, which is an indirect reflection of HIV viral load and activity.[7] Since HIVAN was first described 25 years ago, much has been published regarding the epidemiology, pathogenesis, and treatment of this disease. Despite these advances, however, HIVAN continues to be an impor-tant cause of renal failure.[8] The association between HIV and renal disease was first reported in 1984 by investigators in New York City and Miami, who reported a series of HIV-1–seropositive patients who developed a renal syndrome characterized by progressive renal failure and proteinuria.[1,8] According to the Center for Disease Control, there are approximately 140,000 African Americans currently living with AIDS. These data suggest that there are between 4900 to 17,000 black patients in the United States with HIVAN.[1]

The most common kidney biopsy finding in AIDS has been found to be FSGS-collapsing variant.[1] CG is characterized by black racial predominance, massive proteinuria, relatively rapidly progressive renal insufficiency, and distinctive pathologic findings.[9] CG should be categorized as a separate disease entity because as compared with other variants of FSGS, collapsing FSGS often has global lesions involving >50% of the glomeruli with the highest level of injury to the glomeruli, tubules, and interstitium. The classic pathological findings are of FSGS with focal or global collapsed glomeruli, mesangial matrix deposition, mesangial hyperplasia with increased cellularity due mostly to proliferation of visceral epithelial cells. Visceral epithelial cells show coarse cytoplasmic vacuolization and numerous protein resorption droplets. The presence of even a single collapsed glomerulus clinches the diagnosis.[1,10] Tubular and interstitial inflammation can be prominent and more marked than anticipated for the degree of glomerular injury. Microtubular cystic dilations with casts, a feature that helps distinguish HIVAN from idiopathic CG, are usually prominent and may be directly related to HIV-1 infection of renal epithelial cells. Electron microscopy may reveal tubuloreticular structures in glomerular endothelial cells, which have also been described in lupus nephritis[1] and granulofibrillary transformation of nuclear chromatin in tubular and interstitial cells. Also seen is diffuse effacement of foot processes, less extensive than in minimal change disease. Immunofluorescence microscopy findings are negative or nonspecific.
